# Evidence Center structural and operational conditions and challenges: a descriptive study, Brazil, 2024

**DOI:** 10.1590/S2237-96222026v35e20250457.en

**Published:** 2026-04-19

**Authors:** Y. Juliana Cardozo Amaya, Pedro Henrique Alves Santos, Aurelina Aguiar Lima, Camila Lasse Silva, Fábio Henrique Cavalcanti de Oliveira, Marta Roberta Santana Coelho

**Affiliations:** 1 Ministério da Saúde, Secretaria de Ciência, Tecnologia e Inovação em Saúde, Brasília, DF, Brazil Ministério da Saúde Secretaria de Ciência Tecnologia e Inovação em Saúde Brasília DF Brazil

**Keywords:** Health Policies, Evidence-Informed Policy, Health Management, Health Sciences, Technology and Innovation Management, Health Administration., Políticas de Salud, Política Informada por Evidencia, Gestión en Salud, Gestión de Ciencia, Tecnología y Innovación en Salud, Administración en Salud.

## Abstract

**Objective:**

To describe the physical, structural and operational conditions, as well as the main challenges faced by Evidence Centers (NEvs) recognized by the Evidence-Informed Policy Network (EVIPNet) Brazil.

**Methods:**

This is a descriptive cross-sectional observational study, with data collection carried out between November 2024 and January 2025, using an electronic questionnaire developed based on document analysis and previous studies. The data were analyzed descriptively, presenting absolute and relative frequencies of the investigated variables.

**Results:**

Of the 36 Centers recognized by the EVIPNet Brazil Network, 25 participated in the study (return rate of 69.4%). Among the respondents, 72.0% had their own physical space, 88.0% had computers and 92.0% had access to scientific databases. Their most frequent activities included training (96.0%) and production of technical reports (92.0%). The main challenges reported were: scarcity of financial resources, lack of qualified personnel, infrastructure limitations and difficulties in accessing information.

**Conclusion:**

The structural and operational limitations identified highlight the need for strategies to strengthen the sustainability of the NEvs. Investments in capacity building, institutional coordination and regional equity are fundamental to consolidating evidence-informed public policies in Brazil.

## Resumen

**Objetivo:**

Describir las condiciones físicas, estructurales y operativas, así como los principales desafíos que enfrentan los Centros de Evidencia (NEvs) reconocidos por la Red de Políticas Informadas por la Evidencia (EVIPNet) Brasil.

**Métodos:**

Estudio observacional descriptivo transversal, con recolección de datos realizada entre noviembre de 2024 y enero de 2025, mediante un cuestionario electrónico desarrollado con base en el análisis de documentos y estudios previos. Los datos se analizaron descriptivamente, presentando frecuencias absolutas y relativas de las variables investigadas.

**Resultados:**

De los 36 Centros reconocidos por la Red EVIPNet Brasil, 25 participaron en el estudio (tasa de retorno del 69,4%). Entre los encuestados, el 72,0% contaba con espacio físico propio, el 88,0% con computadoras y el 92,0% con acceso a bases de datos científicas. Las actividades más frecuentes incluyeron capacitación (96,0%) y elaboración de informes técnicos (92,0%). Los principales desafíos reportados fueron: escasez de recursos financieros, falta de personal calificado, limitaciones en la infraestructura y dificultades para acceder a la información.

**Conclusión:**

Las limitaciones estructurales y operativas identificadas resaltan la necesidad de estrategias para fortalecer la sostenibilidad de los NEvs. La inversión en desarrollo de capacidades, coordinación institucional y equidad regional es fundamental para consolidar políticas públicas basadas en la evidencia en Brasil.

**Ethical aspects**

This research used public domain anonymized databases.

## Introduction 

Evidence-Informed Policies (EIPs) for health service provision promote more effective and equitable decisions, reduce repetition of errors, promote rational use of resources and strengthen confidence in policy-making processes ^[^^1^^]^. The Evidence-Informed Policy Network (EVIPNet) initiative, created by the World Health Organization in 2005, aims to produce EIPs, focusing on low- and middle-income countries ^[^^2^ ^-^ ^4^^]^.

In Brazil, the EVIPNet is composed of the Executive Secretariat (part of the Ministry of Health General Coordination of Health Evidence), an advisory council formed by representatives from various Health Ministry Secretariats and other organizations, in addition to the Evidence Centers (*Núcleos de Evidências*, NEvs) and thematic working groups distributed throughout the country ^[^^5^^,^^6^^]^. Established in 2007, the EVIPNet Brazil Network began its activities with 26 institutions and 13 NEvs, expanding progressively. By November 2024, the Network had 36 NEvs distributed across the five regions of the country, demonstrating its commitment to the equitable promotion of the use of evidence in health throughout the national territory ^[^^7^^]^.

The NEvs perform knowledge synthesis and knowledge translation in partnership with decision-makers, which contributes to more equitable, effective and efficient public policies. With their multidisciplinary teams, they act as intermediaries between science and management, aligning evidence production with the demands of the policy cycle, strengthening decision-making and supporting the improvement of the Brazilian Unified Health System (*Sistema Único de Saúde*, SUS) ^[^^8^^]^.

Despite progress made, the sustainability of the Network depends on leadership, governance, funding and monitoring. Since 2019, the General Coordination of Health Evidence has been conducting efforts to strengthen and reorganize the Network through technical support, professional training and definition of consolidation criteria ^[^^9^^]^. In this context, in November 2024, information was gathered on the NEvs, with the aim of mapping, expanding and understanding the structure and interconnections of the EVIPNet Brazil Network.

This study sought to describe the physical, structural and operational conditions, as well as the main challenges faced by the NEvs recognized by EVIPNet Brazil Network in 2024.

## Methods 

Design 

This is a descriptive cross-sectional observational study, based on the collection of self-reported primary data by means of an electronic questionnaire, developed from document analysis and previous studies. The study describes the physical and operational characteristics, as well as the challenges faced by the NEvs recognized by the EVIPNet Brazil Network as at November 2024.

Setting 

The Executive Secretariat of the EVIPNet Brazil Network is responsible for the management and sustainability of the Network, including monitoring the activities of the NEvs, deciding on their inclusion or exclusion, and managing projects funded by the Ministry of Health ^[^^10^^]^. However, the growth of the Network and the need to improve its capacity to support EIPs in different regional contexts highlight the importance of restructuring it, in order to strengthen integration between the NEvs, optimize resources, and increase equity in access to and use of evidence in Brazil.

The NEvs are research groups linked to public health or education institutions, recognized by the EVIPNet Brazil Network since 2017, having the function of supporting EIPs through the synthesis and translation of scientific knowledge. They operate in local Health Departments, hospitals and universities, carrying out evidence syntheses, training and articulation with health service managers. Their members have diverse backgrounds, mainly in health and social sciences, and the requests for studies are made by SUS managers and partner institutions ^[^^9^^]^.

Participants 

All NEvs recognized by EVIPNet Brazil Network were invited to participate. They were required to submit the completed questionnaire about their physical and operational structures between November 27, 2024, and January 15, 2025 (49 days). Each NEv autonomously designated a single representative responsible for completing the questionnaire so as to avoid duplicate responses. This designation was made internally by each Center, following its own organizational criteria, without interference from the research team; generally, it was the NEv coordinator or a member designated by them.

The institutional contacts of these representatives were obtained from Annex I of the Evidence Center Registration Forms, as provided for by the Network’s Internal Regulations ^[10]^, which brought together the updated official information provided by the NEvs. In the event of no response, up to three reminders were sent via institutional email to the registered contact, and no other procedure for replacement or active searching for new respondents was used. It should be noted that only questionnaires submitted within the established deadline were included in the analysis.

Variables

The questionnaire variables analyzed were categorical and descriptive, grouped into six thematic sections, as follows.

Identification: geographic location and institutional affiliation.

Physical structure: available spaces, equipment and access to scientific resources.

Operational structure: team composition and job functions.

Articulation and requests for evidence: relationship with health service managers and processes for receiving and prioritizing requests.

Operation and challenges: funding sources, obstacles faced and support needs.

Suggestions and improvements: proposals made by the teams for improving the structure and functioning of the Centers.

The open-ended responses were manually categorized for the purposes of analysis. There was no imputation of missing data, and all variables were analyzed as received.

Data sources and measurement 

Data collection was carried out between November 2024 and January 2025, using an electronic questionnaire created using Google Forms. Responses to the questionnaire were automatically recorded by the instrument itself. This data was exported to an Excel spreadsheet, which was later downloaded for data analysis. The questionnaire was developed by the team of the EVIPNet Brazil Executive Secretariat based on document analysis of the virtual meeting held on November 27, 2024, in which representatives participated as a data collection strategy.

The questionnaire was built based on an instrument previously used by Oliveira et al. (2020) ^[^^9^^]^ and validated by the General Coordination of Health Evidence, which ensured relevance and suitability to the Network context. Composed of 24 questions - which presented varied formats, such as dichotomous (yes, no), multiple choice, categorical and open-ended - the questionnaire was sent by email to be completed. Periodic reminders were also sent every eight days by email and these were emphasized through direct communication channels, such as Teams and WhatsApp.

Participants submitted their responses voluntarily, after reading and accepting the informed consent record.

Study size

Of the 36 NEvs recognized by EVIPNet Brazil Network and invited to participate, the responses of 25 of them were included in the analysis, as they submitted the questionnaire within the scheduled data collection period. The study was designed as a census, without prior selection of participants; however, the final sample corresponded only to the NEvs that responded on time, characterizing a convenience sample resulting from the response rate. The 11 NEvs that did not submit the instrument, even after reminders, were excluded from the analysis.

Statistical methods

We performed univariate descriptive analysis, with calculation of absolute and relative frequencies for each variable. The percentage participation rates were calculated as follows.

National participation rate: ratio between the number of participating NEvs in each region and the total number of participating NEvs in Brazil.

Regional participation rate: ratio between the number of participating NEvs and the total number of recognized NEvs in each macro-region.

The responses were tabulated and graphically represented using Microsoft Excel 2016 software. For the purposes of visual presentation in the article, the figures were diagrammed using Adobe Illustrator 2025.

## Results 

Of the 36 recognized NEvs, 25 participated in the study, representing 69.4% of the national total. The highest concentration of NEvs and participation related to the Southeast and Northeast regions. The South and Southeast regions presented the highest proportional participation rates, while the North and Northeast regions registered rates equal to or less than 50.0% (Figure 1).

Most NEvs (72.0%) reported having their own physical space, generally larger than 10m², with meeting rooms and suitable environments for collaborative activities (52.0%). Some Centers used shared spaces, which enabled their operation even without exclusive infrastructure (Table 1). The Centers indicated that they had essential equipment, such as computers, printers, scanners, internet connection and office furniture (Table 1).

The most frequent activities performed by NEv professionals were training (96.0%), preparation of technical reports (92.0%) and review of scientific evidence (92.0%) (Figure 2A). These functions were aligned with the institutional activities of the Centers, which included training professionals in the use of evidence (92.0%), promoting use of evidence (92%), disseminating information to health service managers (88.0%), and producing technical notes (84.0%) (Figure 2B).

The NEvs predominantly used free databases, such as PubMed/Medline and the Virtual Health Library (92.0%), to search for evidence. The materials most used in the production of reports were the Methodological Guidelines: Synthesis of Evidence for Policies (96.0%), Virtual Health Library guidelines (84.0%) and portfolios produced by the Ministry of Health (76.0%) (Table 1).

Most NEvs reported that their last three productions, developed in 2024, were used by health service managers in the formulation of EIPs (88.0%). Internal requests for evidence were aligned with the teams’ strategic planning (64.0%), while external requests varied between Centers, with some partially meeting and others prioritizing these requests (Table 1).

**Figure 1:**
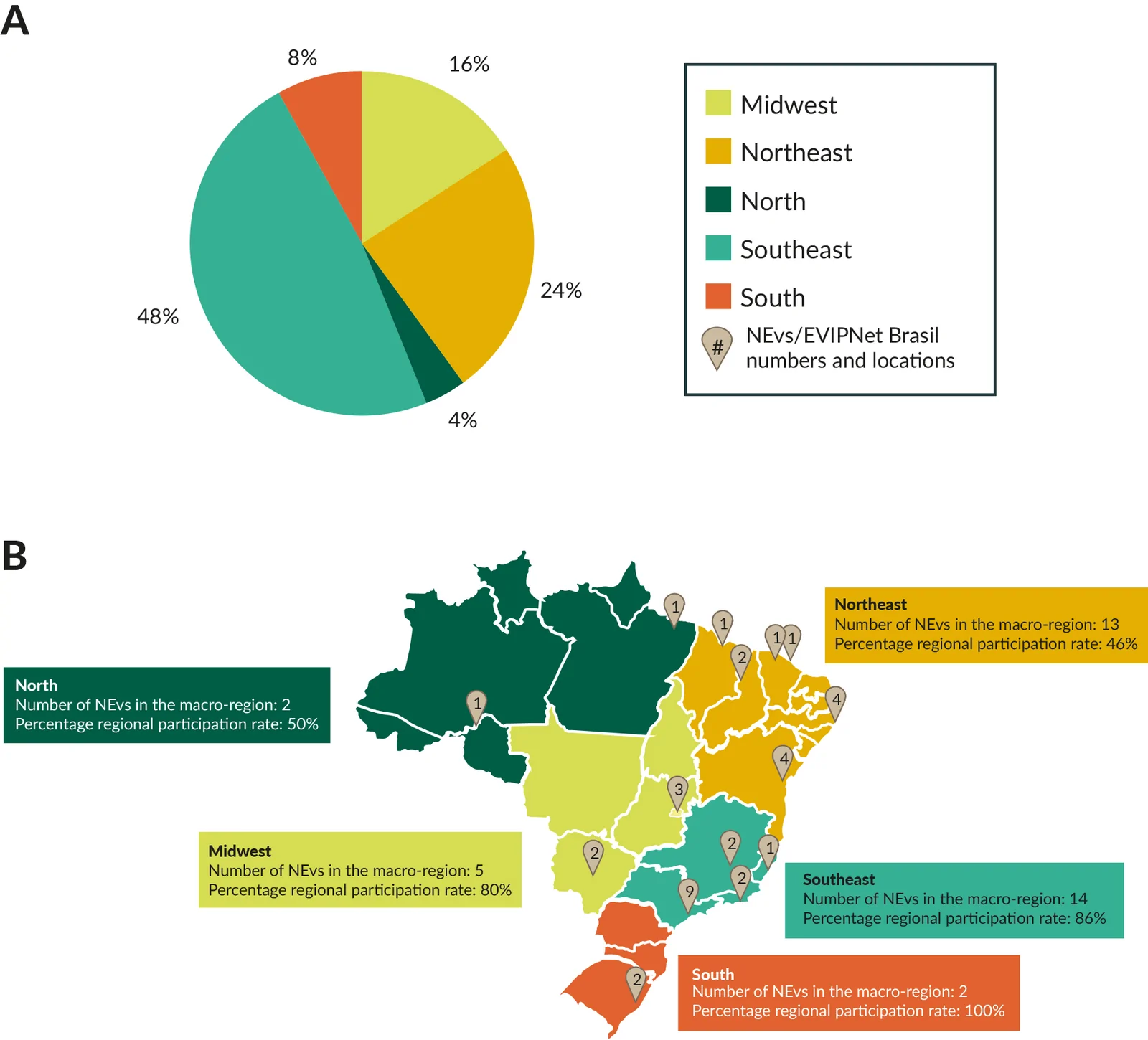
Geographic distribution of the Evidence Centers (NEvs) over the Brazilian regions: (A) national participation rate of the NEvs; (B) proportion of study questionnaire respondents. Brazil, 2024 (n=25)

**Table 1:** Distribution of positive answers given by the Evidence Centers (NEvs) included in the study. Brazil, 2024 (n=25)

Item	Questions	n (%)
1	Does the Center have its own physical space?	
	Yes	18 (72.0)
	No	7 (28.0)
2	If it does have its own physical space, what is the approximate size of the Center’s physical space?	
	Less than 10 m²	4 (16.0)
	Between 10 m² and 20 m²	8 (32.0)
	More than de 20 m²	6 (24.0)
	Not applicable	7 (28.0)
3	If it does have its own physical space, does the Center have a meeting room?	
	Yes	12 (48.0)
	No	6 (24.0)
	Not applicable	7 (28.0)
4	What equipment is available at the NEv?	
	Work desks and chairs	21 (84.0)
	High-speed internet	18 (72.0)
	Scanner	13 (52.0)
	Printer	17 (68.0)
	Computers	22 (88.0)
	Unanswered	2 (8.0)
5	Does the Center have access to scientific databases?	
	Yes	23 (92.0)
	No	2 (8.0)
6	What materials do you use as a basis for production?	
	Guidelines available from the Virtual Health Library	21 (84.0)
	Methodological guidelines: synthesis of evidence for policies	24 (96.0)
	Product portfolio (NEv/Ministry of Health)	19 (76.0)
	Other	4 (16.0)
7	Have any or all three of the productions you mentioned been used by health service managers?	
	Yes	22 (88.0)
	No	2 (8.0)
	Not applicable	1 (4.0)
8	Does the Center articulate with local health service managers?	
	Yes	21 (84.0)
	No	4 (16.0)
9	What are internal requests for evidence based on?	
	Team interest in specific areas	16 (64.0)
	The group’s strategic planning	16 (64.0)
	Other	4 (16.0)
10	Does the Center receive external requests for production of evidence?	
	Yes	21 (84.0)
	No	4 (16.0)
11	If it receives external requests, what percentage do they account for in relation to internal requests?	
	Up to 25%	9 (36.0)
	Between 26% and 50%	3 (12.0)
	Between 51% and 75%	3 (12.0)
	More than 75%	6 (24.0)
12	Does the Center have a structured workflow for prioritizing or meeting external requests?	
	Yes	12 (48.0)
	No	13 (52.0)
13	Does the Center have partnerships with other institutions?	
	Yes	21 (84.0)
	No	4 (16.0)

**Figure 2:**
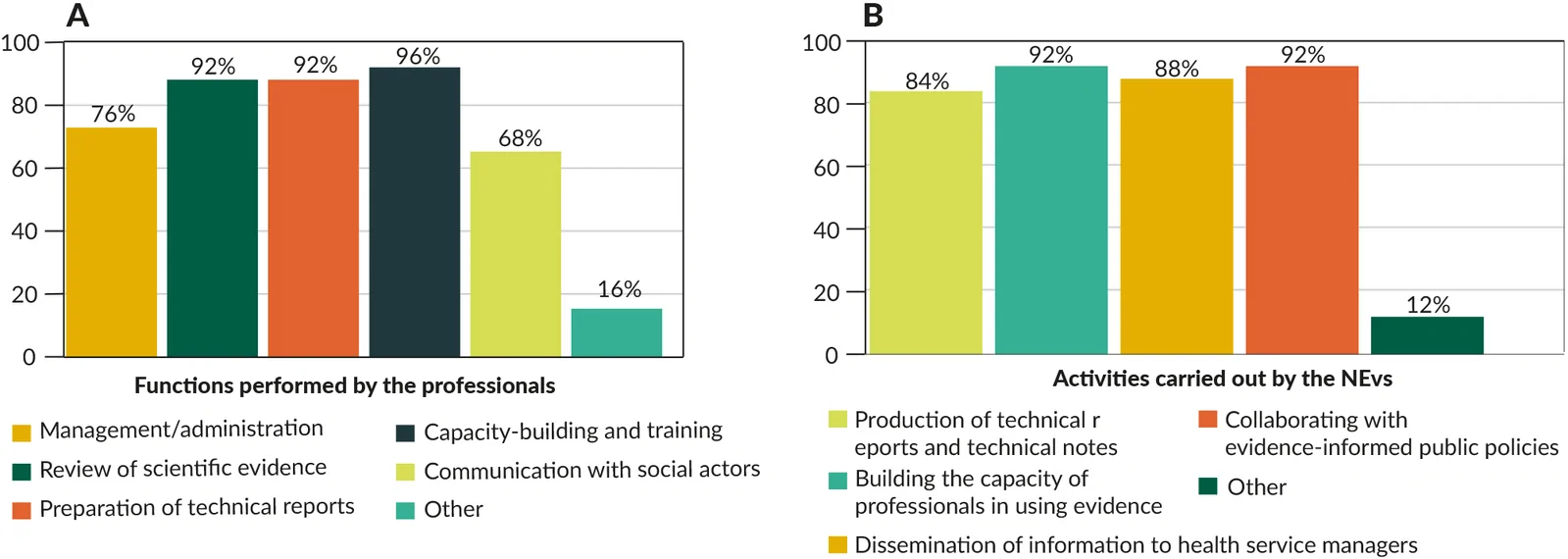
Operational structure of the Evidence Centers (NEvs): (A) functions performed by the health professionals; (B) activities carried out by the NEvs. Brazil, 2024 (n=25)

The main source of NEv funding was taking part in calls to apply for funding (48.0%), followed by institutional resources (28.0%) and, to a lesser extent, municipal resources and service contracts (16.0%). Agreements and partnerships with hospitals, clinics, universities and foundations were also mentioned as fundraising strategies (8.0%) (Figure 3).

Four main challenges were identified: limited financial resources, inadequate infrastructure, shortage of qualified personnel and barriers to accessing information. The latter stemmed from the unavailability of paid platforms and up-to-date data. Other challenges reported included high student turnover, lack of clear rules for managing scholarship grantees, shortage of critical mass among request makers, and the need to strengthen the Network’s structure and local support networks (Figure 4).

Lack of financial resources was the most frequent challenge in all regions. Shortage of qualified personnel was most pronounced in the Northeast, Southeast and Southern regions. In the Midwest, access to information appeared as the second greatest challenge. In the North, the four challenges were reported to be equal, which highlighted the diversity of contexts faced by the NEvs (Figure 4).

**Figure 3:**
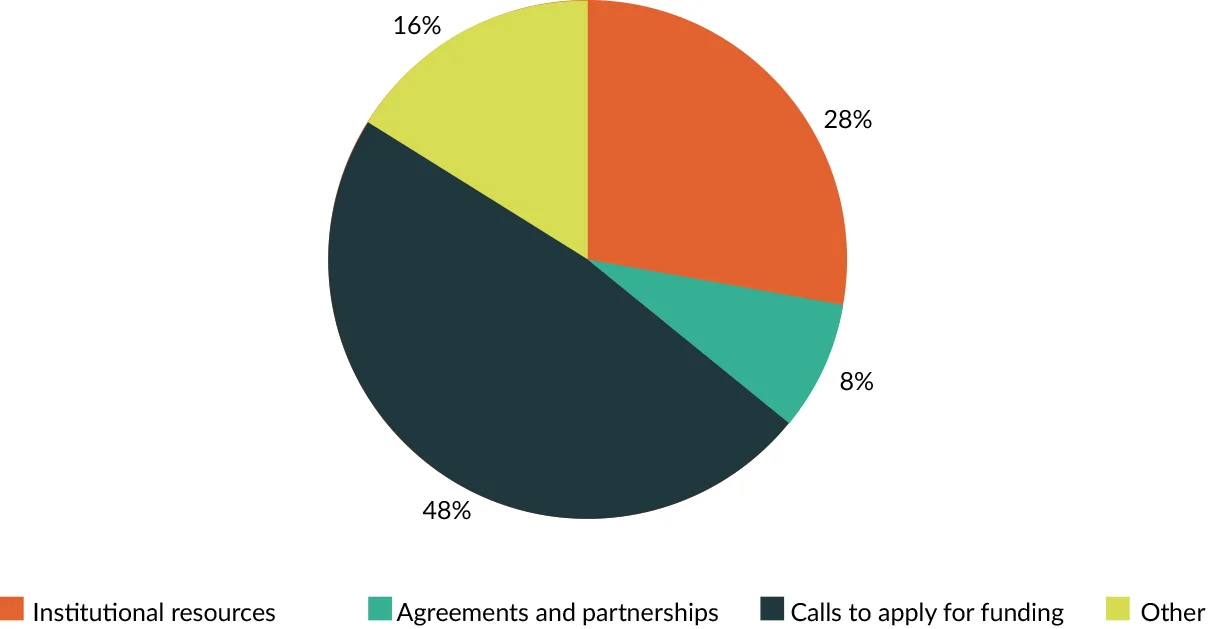
Distribution of the main sources of funding of the Evidence Centers over the Brazilian regions. Brazil, 2024 (n=25)

**Figure 4:**
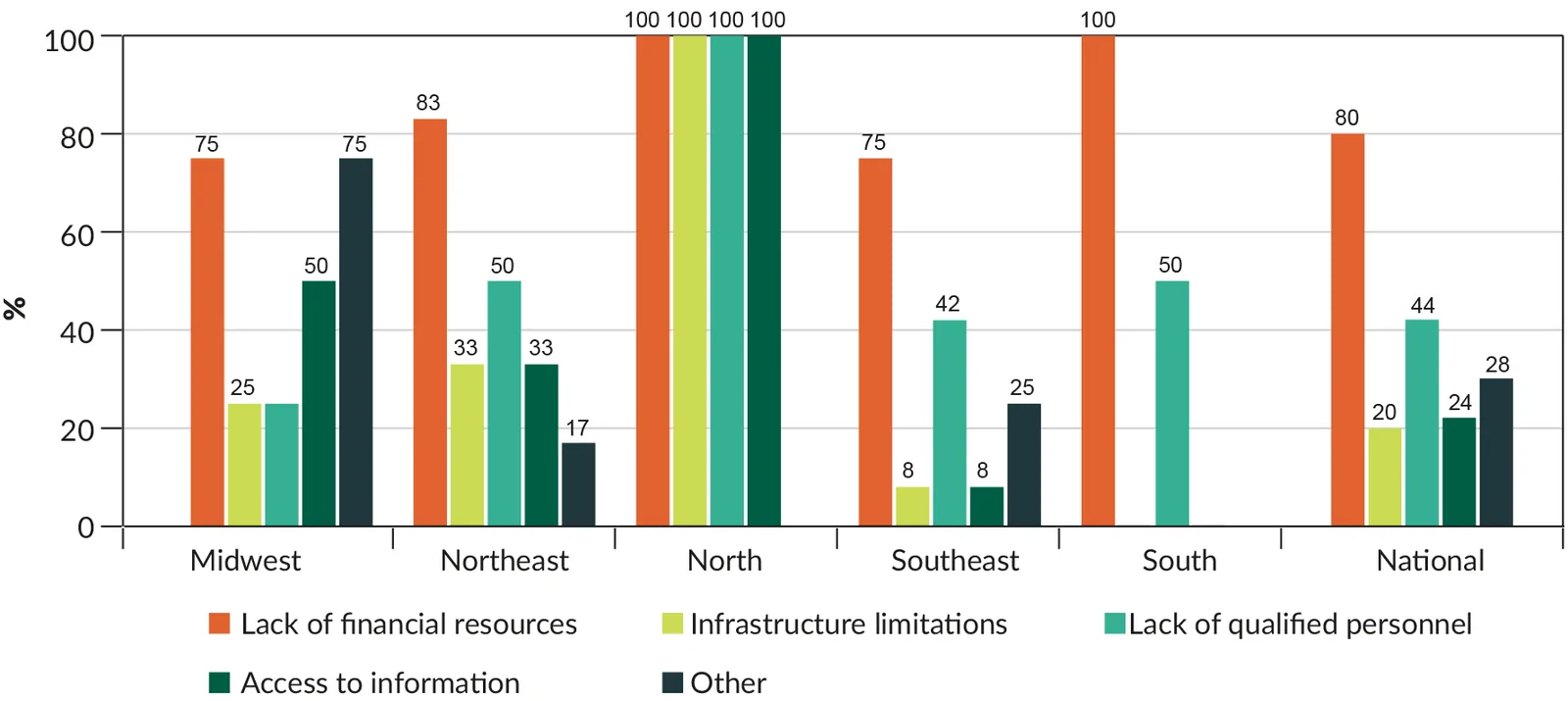
Challenges reported by the Evidence Centers included in the study, by Brazilian region. Brazil, 2024 (n=25)

## Discussion 

The results presented in this study represent the first attempt to recognize the physical and operational characteristics of the EVIPNet Brazil Network, as well as the needs faced by the NEvs that comprise it. Regional inequalities were observed in the distribution and participation of the NEvs, with lower representation in the North and Northeast regions.

Most Centers had their own physical space, internet access and adequate equipment, in addition to conducting training, producing reports and summarizing evidence using free databases, aligning themselves with official decision support instruments. The outputs of the NEvs were used by health service managers, demonstrating local articulation and practical relevance. However, structural and operational challenges persisted, such as funding limitations, infrastructure restrictions, lack of qualified personnel and communication and management difficulties, which directly impacted the sustainability and reach of EIPs in Brazil.

The main limitation of this study was the lower response rate from the NEvs in the North and Northeast regions, which may have compromised the regional and national representativeness of the data. This response bias requires caution when interpreting the results and emphasizes the need to expand the sample in future surveys, ensuring the external validity of the findings and equity in the formulation of EIPs. It should also be noted that the data used reflect self-reported institutional information, which may introduce biases in perception about the infrastructure and performance of the Centers.

The findings of this study are aligned with the literature that points to regional disparities as factors influencing equitable access to evidence for health decision-making ^[^^9^^]^. Implementation of EIPs is subject to political and institutional factors and frequently limited by funding constraints and shortage of human resources, which compromises the sustainability of the NEvs ^[^^9^^,^^11^ ^-^ ^13^^]^.

Institutional capacity varies widely between countries and regions, so that investment in infrastructure and formal mechanisms is essential in order to consolidate the institutionalization of evidence-based decisions and reduce inequalities in access to and use of evidence ^[^^12^^,^^13^^]^. Although the SUS has made progress in reducing regional inequalities ^[^^14^^,^^15^^]^, ensuring equitable evidence-informed decisions across the country continues to be a challenge ^[^^9^^]^.

The infrastructure limitations identified by the NEvs reflect a scenario already pointed out in previous research, in which 56.25% of respondents reported similar difficulties ^[^^16^^]^. The Ministry of Science, Technology and Innovation has stressed the importance of adequate infrastructure for knowledge production and service development ^[^^17^^]^. In this sense, maintaining physical conditions and strengthening technical support are essential for ensuring the sustainability and performance of the NEvs.

These results corroborate studies that highlight the NEvs as key actors in capacity building, network support and knowledge translation, with emphasis on the production of evidence syntheses and provision of training that strengthens decision-making ^[^^7^^,^^9^^]^. The literature also shows the predominant use of free databases such as PubMed/Medline and Virtual Health Library ^[^^18^^,^^19^^]^, which highlights the need for financial support to access paid tools and advanced technologies.

Health service manager use of products developed by NEvs highlights their potential to support the formulation of EIPs ^[^^8^^]^. However, barriers persist between the production and use of knowledge, related to the low capacity for adaptation and application of evidence ^[^^9^^,^^20^^]^. Overcoming these barriers is essential in order to strengthen the institutionalization of EIPs, reduce uncertainties and optimize resources, promoting more participatory and effective decisions in the health system ^[^^11^^,^^12^^,^^21^^,^^22^^]^. Institutionalization of the NEvs increases their legitimacy and priority in taking part in calls for funding proposals, expanding funding opportunities and consolidating their relevance among health service managers, academia and civil society ^[^^8^^]^.

Financial limitations, lack of qualified personnel, infrastructure restrictions and absence of clear management standards are the main challenges faced by the NEvs, which reflect the diversity of regional contexts. These findings converge with research that highlights the importance of clear institutional structures and formal mechanisms to support evidence-informed health policies in different contexts ^[^^20^^]^, as well as with studies that have identified scarcity of financial resources as the greatest obstacle to research ^[^^16^^]^.

Although governance and capacity building have been highlighted as barriers ^[^^9^^,^^16^^]^, initiatives such as the development of guidelines and the internal regulations of the Network point to promising paths for strengthening the Centers. EVIPNet Brazil’s work in qualifying members, through participatory approaches, has contributed to expanding institutional capacity and promoting collective understanding of EIP formulation ^[^^22^^]^. In the context of the Americas, strengthening intersectoral partnerships and investing in evidence-based health systems are indicated as fundamental strategies for reducing inequalities and improving decision-making ^[^^13^^]^.

Strengthening the infrastructure and institutionalization of the NEvs is essential in order to expand their sustainability and impact within the SUS, promoting more effective and equitable health decisions. Specific strategies focused on continuous training, ensuring adequate resources and consolidating governance can contribute to the consolidation of EIPs in Brazil and to building a health system that is more efficient, participatory and responsive to the needs of the population.

This study achieved its objective by characterizing the physical and operational structure of the NEvs recognized by EVIPNet Brazil Network and by identifying factors that influence their functioning. The results provide support for strengthening the Network and improving the Centers’ capacities in the production and use of evidence. Future investigations are recommended so that all the NEvs take part, in order to deepen the understanding of their needs and potential, thus strengthening the use of evidence in health policies.

## Data Availability

The data used in this research are available only upon formal request to the authors for verification purposes, via email to the Executive Secretariat of the EVIPNet Brazil Network (evipnetbrasil@saude.gov.br) or through the contacts available on its website https://brasil.evipnet.org/. The data collection instrument, along with the informed consent record, is publicly available on the OSF platform, accessible at: https://osf.io/d9wug/files/psdfe.
