# Supplementary material for: Evidence Center structural and operational conditions and challenges: a descriptive study, Brazil, 2024
Source: Epidemiol Serv Saude. 2026 Apr 19;35:e20250457. doi: 10.1590/S2237-96222026v35e20250457.en (PMC13686376; doi:10.1590/S2237-96222026v35e20250457.en)
Supplement: Translation

# Translation: Condições e desafios estruturais e operacionais dos núcleos de evidência: estudo observacional descritivo transversal, Brasil, 2024

**DOI:** 10.1590/S2237-96222026v35e20250457.pt

**Authors:** Y. Juliana Cardozo Amaya, Pedro Henrique Alves Santos, Aurelina Aguiar Lima, Camila Lasse Silva, Fábio Henrique Cavalcanti de Oliveira, Marta Roberta Santana Coelho

**Affiliations:** 1 Ministério da Saúde, Secretaria de Ciência, Tecnologia e Inovação em Saúde, Brasília, DF, Brasil Ministério da Saúde Secretaria de Ciência Tecnologia e Inovação em Saúde Brasília DF Brasil

**Keywords:** Políticas de Saúde, Política Informada por Evidências, Gestão em Saúde, Gestão de Ciência, Tecnologia e Inovação em Saúde, Administração em Saúde.

## Abstract

**Objetivo:**

Descrever as condições físicas, estruturais e operacionais, além dos principais desafios enfrentados pelos núcleos de evidência reconhecidos pela Evidence-Informed Policy Network (EVIPNet) Brasil.

**Métodos:**

Estudo observacional descritivo transversal, com coleta de dados realizada entre novembro de 2024 e janeiro de 2025, por meio de questionário eletrônico, elaborado com base em análise documental e estudos prévios. Os dados foram analisados de forma descritiva, com apresentação de frequências absolutas e relativas das variáveis investigadas.

**Resultados:**

Dos 36 núcleos reconhecidos pela Rede EVIPNet Brasil, 25 participaram do estudo (taxa de retorno de 69,4%). Entre os respondentes, 72,0% possuíam espaço físico próprio, 88,0% dispunham de computadores e 92,0% tinham acesso a bases científicas. As atividades mais frequentes incluíam capacitações (96,0%) e produção de relatórios técnicos (92,0%). Os principais desafios relatados foram: escassez de recursos financeiros, carência de pessoal qualificado, limitações na infraestrutura e dificuldades de acesso à informação.

**Conclusão:**

As limitações estruturais e operacionais identificadas evidenciam a necessidade de estratégias para fortalecer a sustentabilidade dos NEv. Investimentos em capacitação, articulação institucional e equidade regional são fundamentais para consolidar políticas públicas informadas por evidências no Brasil.

**Aspectos éticos**

A pesquisa utilizou banco de dados de domínio público e anonimizados.

## Introdução 

As Políticas Informadas por Evidências (PIE) na saúde favorecem decisões mais eficazes e equitativas, reduzem a repetição de erros, promovem o uso racional de recursos e fortalecem a confiança nos processos de formulação de políticas ^[^^1^^]^. A iniciativa Evidence-Informed Policy Network (EVIPNet), criada pela Organização Mundial da Saúde em 2005, visa à produção de PIE, com foco em países de baixa e média renda ^[^^2^ ^-^ ^4^^]^.

No Brasil, a EVIPNet é composta pela Secretaria-Executiva (vinculada à Coordenação-Geral de Evidências em Saúde do Ministério da Saúde), por um conselho consultivo formado por representantes de diversas secretarias do Ministério e de outras organizações, além dos núcleos de evidências (NEv) e dos grupos de trabalho temáticos distribuídos pelo país ^[^^5^^,^^6^^]^. Instituída em 2007, a Rede EVIPNet Brasil iniciou suas atividades com 26 instituições e 13 NEv, expandindo-se progressivamente. Até novembro de 2024, a Rede contava com 36 NEv distribuídos pelas cinco regiões do país, evidenciando seu compromisso com a promoção equitativa do uso de evidências em saúde no território nacional ^[^^7^^]^.

Os NEv realizam sínteses e tradução do conhecimento em parceria com tomadores de decisão, o que contribui para políticas públicas mais equitativas, efetivas e eficientes. Com equipes multidisciplinares, atuam como intermediários entre ciência e gestão, alinhando a produção de evidências às demandas do ciclo de políticas, fortalecendo a tomada de decisão e apoiando o aprimoramento do Sistema Único de Saúde (SUS) no Brasil ^[^^8^^]^.

Apesar dos avanços, a sustentabilidade da Rede depende de liderança, governança, financiamento e monitoramento. Desde 2019, a Coordenação-Geral de Evidências em Saúde tem conduzido esforços para fortalecer e reorganizar a Rede por meio de apoio técnico, capacitação profissional e definição de critérios de consolidação ^[^^9^^]^. Nesse contexto, em novembro de 2024, foram levantadas informações sobre os NEv, com o objetivo de mapear, ampliar e compreender a estrutura e as interconexões da Rede EVIPNet Brasil. 

Este estudo buscou descrever as condições físicas, estruturais e operacionais, além dos principais desafios enfrentados pelos NEv reconhecidos pela EVIPNet Brasil em 2024.

## Métodos 

Delineamento 

Trata-se de estudo observacional descritivo transversal, com base na coleta de dados primários autorrelatados por meio de questionário eletrônico, elaborado a partir de análise documental e estudos prévios. O estudo descreve as características físicas e operacionais, bem como os desafios enfrentados pelos NEv reconhecidos pela Rede EVIPNet Brasil, até novembro de 2024.

Contexto 

A Secretaria-Executiva da Rede EVIPNet Brasil é responsável pela gestão e sustentabilidade da Rede, incluindo o monitoramento das atividades dos NEv, a deliberação sobre sua inclusão ou desligamento e a gestão de projetos financiados pelo Ministério da Saúde ^[^^10^^]^. Entretanto, o crescimento da Rede e a necessidade de aprimorar a sua capacidade de apoiar PIE em diferentes contextos regionais evidenciam a importância de sua reestruturação, de forma a fortalecer a integração entre os NEv, otimizar recursos e ampliar a equidade no acesso e uso de evidências no país.

Os NEv são grupos de pesquisa vinculados a instituições públicas de saúde ou ensino, reconhecidos pela EVIPNet Brasil desde 2017, com a função de apoiar PIE por meio da síntese e tradução do conhecimento científico. Atuam em secretarias de saúde, hospitais e universidades, realizando sínteses de evidências, capacitações e articulação com gestores. Seus membros possuem formações diversas, principalmente em saúde e ciências sociais, e as demandas de estudos são solicitadas por gestores do SUS e instituições parceiras ^[^^9^^]^.

Participantes 

Foram convidados a participar todos os NEv reconhecidos pela EVIPNet Brasil. Estes deveriam enviar o questionário sobre suas estruturas física e operacional devidamente preenchido entre 27 de novembro de 2024 e 15 de janeiro de 2025 (49 dias). Cada NEv indicou, de forma autônoma, um único representante responsável pelo preenchimento, com a finalidade de evitar duplicidade de respostas. Essa indicação foi realizada internamente por cada núcleo, seguindo seus próprios critérios organizacionais, sem interferência da equipe de pesquisa; geralmente, tratava-se do coordenador do NEv ou de um membro por ele designado. 

Os contatos institucionais desses representantes foram obtidos a partir do Anexo I - Ficha de Cadastro do Núcleo de Evidências, constante no Regimento Interno da Rede ^[^^10^^]^ que reunia as informações oficiais atualizadas fornecidas pelos próprios NEv. Em caso de ausência de resposta, foram realizados até três lembretes por e-mail institucional ao contato cadastrado, não sendo utilizado outro procedimento de substituição ou busca ativa de novos respondentes. Ressalta-se que apenas os questionários enviados no prazo estabelecido foram considerados na análise.

Variáveis

As variáveis analisadas no questionário foram categóricas e descritivas, agrupadas em seis seções temáticas, conforme a seguir.

Identificação: localização geográfica e vínculo institucional.

Estrutura física: espaços disponíveis, equipamentos e acesso a bases científicas.

Estrutura operacional: composição das equipes e funções desempenhadas.

Articulação e demandas: relacionamento com gestores e processos de recebimento e priorização de demandas.

Funcionamento e desafios: fontes de financiamento, obstáculos enfrentados e necessidades de suporte.

Sugestões e melhorias: propostas das equipes para aprimorar a estrutura e o funcionamento dos núcleos.

As respostas abertas foram categorizadas manualmente para fins de análise. Não houve imputação de dados faltantes, e todas as variáveis foram analisadas conforme recebidas.

Fontes de dados/mensuração 

A coleta de dados foi realizada entre novembro de 2024 e janeiro de 2025, por meio de questionário eletrônico elaborado no Google Forms. As respostas ao questionário foram registradas automaticamente pelo próprio instrumento; disso, foi gerada uma planilha em Excel, posteriormente baixada para a análise dos dados. O instrumento foi desenvolvido pela equipe da Secretaria-Executiva da EVIPNet Brasil com base em análise documental da reunião virtual realizada em 27 de novembro de 2024, da qual participaram representantes de 22 NEv. Esses representantes concordaram com a elaboração e o envio do instrumento como estratégia de coleta de dados.

O questionário foi construído com base em instrumento previamente utilizado por Oliveira et al. (2020) ^[^^9^^]^ e validado pela Coordenação-Geral de Evidências em Saúde, o que garantiu relevância e adequação ao contexto da Rede. Composto por 24 perguntas - as quais apresentaram formatos variados, como dicotômicas (sim, não), de múltipla escolha, categóricas e dissertativas -, o questionário foi enviado por e-mail para que fosse preenchido. Também foram enviados lembretes periódicos a cada oito dias por e-mail e reforço por canais de comunicação direta, como Teams e WhatsApp.

Os participantes encaminharam suas respostas de forma voluntária, mediante leitura e aceite do registro de consentimento livre e esclarecido. 

Tamanho do estudo

Dos 36 NEv reconhecidos pela EVIPNet Brasil e convidados a participar, 25 tiveram suas respostas incluídas na análise, pois enviaram o questionário no período previsto para a coleta. O estudo foi concebido como um censo, sem seleção prévia dos participantes; contudo, a amostra final correspondeu apenas aos NEv que responderam no prazo, caracterizando uma amostra de conveniência resultante da taxa de resposta. Os 11 NEv que não enviaram o instrumento, mesmo após os lembretes, foram excluídos da análise.

Métodos estatísticos

Realizou-se análise descritiva univariada, com cálculo de frequências absolutas e relativas para cada variável. Foram calculadas as porcentagens de participação, conforme a seguir.

Participação nacional: razão entre o número de NEv participantes em cada região e o total de NEv participantes no Brasil.

Participação regional: razão entre o número de NEv participantes e o total de NEv reconhecidos em cada macrorregião.

As respostas foram tabuladas e representadas graficamente por meio do software Microsoft Excel 2016. Para fins de apresentação visual no artigo, as figuras foram diagramadas no Adobe Illustrator 2025.

## Resultados 

Dos 36 NEv reconhecidos, 25 participaram do estudo, o que representou 69,4% do total nacional. A maior concentração de NEv e de participação foi observada no Sudeste e no Nordeste. As regiões Sul e Sudeste apresentaram as maiores taxas de participação proporcional, enquanto Norte e Nordeste registraram taxas iguais ou inferiores a 50,0% (Figura 1).

A maioria dos NEv (72,0%) relatou dispor de espaço físico próprio, geralmente superior a 10 m², com salas de reunião e ambientes adequados para atividades colaborativas (52,0%). Alguns núcleos utilizam espaços compartilhados, o que viabilizou suas operações mesmo sem infraestrutura exclusiva (Tabela 1). Os núcleos indicaram possuir equipamentos essenciais, como computadores, impressoras, scanners, conexão de internet e mobiliário (Tabela 1). 

As atividades mais frequentes realizadas pelos profissionais dos NEv foram capacitações (96,0%), elaboração de relatórios técnicos (92,0%) e revisão de evidências científicas (92,0%) (Figura 2A). Essas funções estavam alinhadas às atividades institucionais dos núcleos, que incluíram capacitação de profissionais em uso de evidências (92,0%), promoção do uso de evidências (92%), disseminação de informações para gestores (88,0%) e produção de notas técnicas (84,0%) (Figura 2B).

Na busca por evidências, os NEv utilizaram predominantemente bases gratuitas, como PubMed/Medline e Biblioteca Virtual em Saúde (92,0%). Os materiais mais empregados na produção de relatórios foram a Diretriz Metodológica: Síntese de Evidências para Políticas (96,0%), diretrizes da Biblioteca Virtual em Saúde (84,0%) e portfólios produzidos pelo Ministério da Saúde (76,0%) (Tabela 1).

A maioria dos NEv relatou que suas três últimas produções, desenvolvidas em 2024, foram utilizadas por gestores na formulação de PIE (88,0%). As demandas internas estavam alinhadas ao planejamento estratégico das equipes (64,0%), enquanto as demandas externas variavam entre núcleos, com alguns atendendo parcialmente e outros priorizando essas solicitações (Tabela 1).

**Figura 1:** Distribuição geográfica dos núcleos de evidências (NEv) pelas regiões brasileiras: (A) participação nacional dos NEv; (B) proporção dos respondentes ao questionário do estudo. Brasil, 2024 (n=25)

**Tabela 1:** Distribuição de respostas positivas dos núcleos de evidências (NEv) incluídos no estudo. Brasil, 2024 (n=25)

Item	Questões	n (%)
1	O núcleo possui um espaço físico próprio?	
	Sim	18 (72,0)
	Não	7 (28,0)
2	Caso possua espaço físico, qual o tamanho aproximado do espaço físico do núcleo?	
	Menos de 10 m²	4 (16,0)
	Entre 10 m² e 20 m²	8 (32,0)
	Mais de 20 m²	6 (24,0)
	Não aplicável	7 (28,0)
3	Caso possua espaço físico, o núcleo possui sala de reunião?	
	Sim	12 (48,0)
	Não	6 (24,0)
	Não aplicável	7 (28,0)
4	Quais são os equipamentos disponíveis no NEv?	
	Mesas e cadeiras para trabalho	21 (84,0)
	Internet de alta velocidade	18 (72,0)
	Scanner	13 (52,0)
	Impressora	17 (68,0)
	Computadores	22 (88,0)
	Não respondido	2 (8,0)
5	O núcleo possui acesso a bases de dados científicas?	
	Sim	23 (92,0)
	Não	2 (8,0)
6	Quais materiais utilizam como base para a produção?	
	Diretrizes disponíveis na Biblioteca Virtual em Saúde	21 (84,0)
	Diretriz metodológica: síntese de evidências para políticas	24 (96,0)
	Portfólio de produtos (NEv/Ministério da Saúde)	19 (76,0)
	Outros	4 (16,0)
7	Algumas ou todas as três produções informadas foram utilizadas por gestores?	
	Sim	22 (88,0)
	Não	2 (8,0)
	Não aplicável	1 (4,0)
8	O núcleo se articula com gestores locais?	
	Sim	21 (84,0)
	Não	4 (16,0)
9	Em que se baseiam as demandas internas?	
	Interesse da equipe em áreas específicas	16 (64,0)
	Planejamento estratégico do grupo	16 (64,0)
	Outras	4 (16,0)
10	O núcleo recebe demandas externas para a produção de evidências?	
	Sim	21 (84,0)
	Não	4 (16,0)
11	Caso receba demandas externas, qual a proporção aproximada em relação às demandas internas?	
	Até 25%	9 (36,0)
	Entre 26% e 50%	3 (12,0)
	Entre 51% e 75%	3 (12,0)
	Mais de 75%	6 (24,0)
12	O núcleo possui um fluxo estruturado para priorizar ou responder a demandas externas?	
	Sim	12 (48,0)
	Não	13 (52,0)
13	O núcleo possui parcerias com outras instituições?	
	Sim	21 (84,0)
	Não	4 (16,0)

**Figura 2:** Estrutura operacional dos núcleos de evidências (NEv): (A) funções desempenhadas pelos profissionais; (B) atividades realizadas. Brasil, 2024 (n=25)

A principal fonte de financiamento dos NEv foi a participação em editais de fomento (48,0%), seguida por recursos institucionais (28,0%) e, em menor proporção, por recursos municipais e contratos de serviços (16,0%). Convênios e parcerias com hospitais, clínicas, universidades e fundações também foram mencionados como estratégias de captação de recursos (8,0%) (Figura 3).

Quatro desafios principais foram identificados: limitação de recursos financeiros, infraestrutura inadequada, escassez de pessoal qualificado e barreiras no acesso à informação. Esse último decorreu da indisponibilidade de plataformas pagas e de dados atualizados. Outros desafios relatados incluíram alta rotatividade de estudantes, ausência de regras claras para gestão de bolsistas, escassez de massa crítica entre os demandantes e necessidade de fortalecer a estrutura da Rede e de redes locais de apoio (Figura 4).

A falta de recursos financeiros foi o desafio mais frequente em todas as regiões. A carência de pessoal qualificado foi mais expressiva nas regiões Nordeste, Sudeste e Sul. No Centro-Oeste, o acesso à informação apareceu como o segundo maior desafio. No Norte, os quatro desafios foram relatados de forma equilibrada, o que evidenciou a diversidade de contextos enfrentados pelos NEv (Figura 4).

**Figura 3:** Distribuição das principais fontes de financiamento dos núcleos de evidências pelas regiões brasileiras. Brasil, 2024 (n=25)

**Figura 4:** Desafios relatados pelos núcleos de evidências incluídos no estudo, conforme as regiões brasileiras. Brasil, 2024 (n=25)

## Discussão 

Os resultados apresentados neste estudo representaram a primeira aproximação ao reconhecimento das características físicas e operacionais da Rede EVIPNet Brasil, bem como das necessidades enfrentadas pelos NEv que a compõem. Observou-se a presença de desigualdades regionais na distribuição e participação dos NEv, com menor representação nas regiões Norte e Nordeste. 

A maioria dos núcleos possuía espaço físico próprio, acesso à internet e equipamentos adequados, além de realizar capacitações, relatórios e sínteses de evidências com o uso de bases gratuitas, alinhando-se a instrumentos oficiais de apoio à decisão. As produções dos NEv foram utilizadas por gestores, o que demonstrou articulação local e relevância prática. Entretanto, persistiram desafios estruturais e operacionais, como limitações de financiamento, restrições de infraestrutura, carência de pessoal qualificado e dificuldades de comunicação e gestão, que impactaram diretamente a sustentabilidade e o alcance das PIE no país.

A principal limitação deste estudo referiu-se à menor taxa de resposta dos NEv das regiões Norte e Nordeste, o que pode ter comprometido a representatividade regional e nacional dos dados. Esse viés de resposta exige cautela na interpretação dos resultados e reforça a necessidade de ampliar a amostra em futuros levantamentos, assegurando a validade externa dos achados e a equidade na formulação de PIE. Ressalta-se ainda que os dados utilizados refletem informações institucionais autorreferidas, o que pode introduzir vieses de percepção sobre a infraestrutura e o desempenho dos núcleos.

Os achados deste estudo estão alinhados à literatura que aponta as disparidades regionais como fatores que influenciam o acesso equitativo às evidências para decisões em saúde ^[^^9^^]^. A implementação das PIE é condicionada por fatores políticos e institucionais e frequentemente limitada por restrições de financiamento e escassez de recursos humanos, o que compromete a sustentabilidade dos NEv ^[^^9^^,^^11^ ^-^ ^13^^]^. 

A capacidade institucional varia amplamente entre países e regiões, tornando essencial o investimento em infraestrutura e mecanismos formais que consolidem a institucionalização das PIE e reduzam desigualdades no acesso e uso de evidências ^[^^12^^,^^13^^]^. Embora o SUS tenha avançado na redução das desigualdades regionais ^[^^14^^,^^15^^]^, garantir decisões informadas por evidências de forma equitativa em todo o território nacional ainda é um desafio ^[^^9^^]^.

As limitações de infraestrutura identificadas pelos NEv refletem um cenário já apontado em pesquisas anteriores, em que 56,25% dos entrevistados relataram dificuldades semelhantes ^[16]^. O Ministério da Ciência, Tecnologia e Inovação reforçou a importância de infraestrutura adequada para a produção de conhecimento e desenvolvimento de serviços ^[^^17^^]^. Nesse sentido, a manutenção das condições físicas e o fortalecimento do suporte técnico são essenciais para assegurar a sustentabilidade e o desempenho dos NEv. 

Estes resultados corroboram estudos que destacam os NEv como atores centrais na capacitação, suporte à rede e tradução do conhecimento, com ênfase na produção de sínteses de evidências e na realização de treinamentos que fortalecem a tomada de decisão ^[^^7^^,^^9^^]^. A literatura também evidencia o uso predominante de bases gratuitas como PubMed/Medline e Biblioteca Virtual em Saúde ^[18,19]^, o que reforça a necessidade de apoio financeiro para acessar ferramentas pagas e tecnologias avançadas.

A utilização dos produtos desenvolvidos pelos NEv por gestores reforça seu potencial para apoiar a formulação de PIE ^[^^8^^]^. Contudo, persistem barreiras entre a produção e o uso do conhecimento, relacionadas à baixa capacidade de adaptação e aplicação das evidências ^[^^9^^,^^20^^]^. Superar essas barreiras é primordial para fortalecer a institucionalização das PIE, reduzir incertezas e otimizar recursos, promovendo decisões mais participativas e eficazes no sistema de saúde ^[^^11^^,^^12^^,^^21^^,^^22^^]^. A institucionalização dos NEv aumenta sua legitimidade e prioridade em editais de fomento, ampliando as oportunidades de financiamento e consolidando sua relevância junto a gestores, academia e sociedade civil ^[8]^.

Limitações financeiras, carência de pessoal qualificado, restrições de infraestrutura e ausência de normas claras de gestão são os principais desafios enfrentados pelos NEv, os quais refletem a diversidade de contextos regionais. Esses achados convergem com pesquisas que ressaltam a importância de estruturas institucionais claras e mecanismos formais para sustentar políticas de saúde informadas por evidências em diferentes contextos ^[^^20^^]^, bem como com estudos que identificaram a escassez de recursos financeiros como o maior obstáculo à pesquisa ^[^^16^^]^. 

Embora a governança e a capacitação tenham sido destacadas como barreiras ^[^^9^^,^^16^^]^, iniciativas como a elaboração de diretrizes e o regimento interno da Rede apontam caminhos promissores para o fortalecimento dos núcleos. A atuação da EVIPNet Brasil na qualificação dos membros, por meio de abordagens participativas, tem contribuído para ampliar a capacidade institucional e promover o entendimento coletivo sobre a formulação de PIE ^[^^22^^]^. No contexto das Américas, o fortalecimento das parcerias intersetoriais e o investimento em sistemas de saúde com base em evidências são indicados como estratégias fundamentais para reduzir desigualdades e aprimorar a tomada de decisão ^[^^13^^]^.

Fortalecer a infraestrutura e a institucionalização dos NEv é essencial para ampliar sua sustentabilidade e impacto no SUS, promovendo decisões em saúde mais eficazes e equitativas. Estratégias específicas voltadas à capacitação contínua, à garantia de recursos adequados e à consolidação da governança podem contribuir para a consolidação das PIE no país e para a construção de um sistema de saúde mais eficiente, participativo e responsivo às demandas da população.

Este estudo alcançou seu objetivo ao caracterizar a estrutura física e operacional dos NEv reconhecidos pela EVIPNet Brasil e ao identificar fatores que influenciam seu funcionamento. Os resultados oferecem subsídios para fortalecer a Rede e para aprimorar as capacidades dos núcleos na produção e no uso de evidências. Recomenda-se a realização de investigações futuras que ampliem a participação de todos os NEv, de modo a aprofundar a compreensão sobre suas necessidades e potencialidades, fortalecendo a utilização de evidências nas políticas de saúde.

## Data Availability

Os dados utilizados na pesquisa estão disponíveis apenas mediante solicitação formal aos autores, para fins de verificação, via e-mail da Secretaria-Executiva da Rede EVIPNet Brasil (evipnetbrasil@saude.gov.br) ou por meio dos contatos disponíveis no site https://brasil.evipnet.org/. O instrumento de coleta de dados, acompanhado do registro de consentimento livre e esclarecido, está disponibilizado para acesso público na plataforma OSF, disponível em: https://osf.io/d9wug/files/psdfe.
